# Clinical Approach to Assessment and Amelioration of Atherosclerotic Vascular Disease in Diabetes

**DOI:** 10.3389/fcvm.2020.582826

**Published:** 2020-10-06

**Authors:** Ronald B. Goldberg

**Affiliations:** Division of Endocrinology, Diabetes and Metabolism, Department of Medicine, Diabetes Research Institute, University of Miami Miller School of Medicine, Miami, FL, United States

**Keywords:** diabetes, cardiovascular disease, risk assessment, prevention, clinical trials

## Abstract

Atherosclerotic cardiovascular disease is increased on average 2–3-fold in people with diabetes as compared to their non-diabetic counterparts and is the major cause of the increased morbidity and mortality in this disease. There is however heterogeneity in cardiovascular risk between individuals based on demographic, cardiometabolic and clinical risk factors in the setting of hyperglycemia, insulin resistance and obesity that needs to be taken into consideration in planning preventive interventions. Randomized clinical trials of agents or procedures used for amelioration of augmented CVD risk in diabetes have been pivotal in providing evidenced-based treatments. Improvement in hyperglycemia in both type 1 and type 2 diabetes is considered to be central in the prevention of microvascular and macrovascular complications although selected antihyperglycemic agents have demonstrated beneficial as well as possible deleterious off-target effects. Lowering low density lipoprotein cholesterol, treating hypertension and stopping smoking each play important roles in preventing cardiovascular disease in diabetes as they do in the general population and low dose aspirin is overall beneficial in high risk individuals. Hypertriglyceridemia may represent another important marker for augmented cardiovascular risk in diabetes and newer agents targeting dyslipidemia appear promising. The fall in cardiovascular events over the past two decades offers hope that modern intervention strategies as well as novel approaches such as those targeting inflammation may contribute to a continued reduction of cardiovascular disease in people with diabetes.

## Introduction

It has been recognized for decades that people with diabetes have an increased risk for atherosclerotic vascular disease (ASCVD). The Framingham Study was one of the early studies that reported that cardiovascular disease (CVD) events in those with diabetes was increased 3-fold in men and 4-fold in women (1). Coronary heart disease (CHD) rates were double in men and 3 times higher in women with diabetes than their non-diabetic counterparts, with similar excess rates for stroke except that that these sex differences were reversed. Event rates for peripheral vascular disease and heart failure (HF) were increased even more, especially in women (8–10-fold). It has become clear that ASCVD is the leading cause of morbidity and mortality in diabetes and its health and economic burden has grown with the epidemics of obesity and diabetes. Furthermore, it has become clear that while augmented atherosclerosis is the major factor underlying the high rates of CVD in diabetes, structural and functional abnormalities of cardiac muscle and its autonomic innervation have a major influence on morbidity and mortality, particularly in older people (2). As a consequence, understanding the nature of CVD and developing strategies for its prevention and treatment in people with diabetes has become a priority.

## Heterogeneity in the Risk for ASCVD in Diabetes

In 2001, the National Cholesterol Education Panel in its Adult Treatment Panel III guidelines recommended that adults with diabetes and without CVD be considered a CHD risk equivalent, assigning a 10 year ASCVD risk of at least 20% (3). However, it subsequently became evident that while this may be true in older people with long-standing diabetes (4) there is significant heterogeneity of risk for ASCVD in people with diabetes (5, 6). Among key determinants of risk are demographic factors such as age, sex, race/ethnicity, and socioeconomic status, duration and type of diabetes, and the number and severity of major risk factors including hyperglycemia itself, as well as risk enhancers, some of which are specific to diabetes and others that are not (Table 1). It is also likely that genetic factors play an important role. How these factors interact to accelerate atherosclerosis in diabetes is incompletely understood.

**Table 1:** Known associations between demographic, clinical and cardiometabolic risk factors and increased atherosclerotic cardiovascular disease (ASCVD) risk in diabetes.

**Factor**	**Direction of association with ASCVD risk**
Demographic	
Age	Increased
Sex	Women have a greater increase in relative risk; men have a greater increase in absolute risk
Race/Ethnicity	South Asians have greater risk
Socioeconomic	Increased in lower socioeconomic groups
Duration of diabetes	Increased
Major risk factors	
LDL-C	Increased with no apparent threshold for risk
Hypertension	Increased from a systolic blood pressure of 120 mm Hg
Smoking	Increased
HDL-C	Decreased in population studies, but HDL function may be a better risk factor
Hyperglycemia	Increases risk but studies are confounded by off-target effects of anti-hyperglycemic agents; findings clearest in type 1 diabetes
Insulin resistance	Increased
Dyslipidemia	Hypertriglyceridemia associated with increased risk
Risk enhancers	Increased (See Table 2 for list)

*LDL-C, low density lipoprotein cholesterol; HDL-C, high density lipoprotein cholesterol*.

### Pathophysiologic Issues

The central, clinically relevant pathophysiologic abnormalities in diabetes are hyperglycemia, insulin deficiency and insulin resistance and the accompanying alterations in metabolic fluxes. While hyperglycemia defines diabetes, varying only in severity, insulin resistance coupled with defective insulin secretion is typically found in type 2 diabetes whereas type 1 diabetes is caused by severe insulin deficiency. Obesity which is linked to the development of type 2 diabetes, is a major determinant of insulin resistance. Obesity is also increasingly being recognized as a feature of type 1 diabetes as intensive insulinization is often associated with weight gain. It is the interplay of hyperglycemia and insulin resistance and the accompanying metabolic alterations complicated by obesity that is thought to drive oxidative stress, subclinical inflammation, and a procoagulant state, which leads to the functional and structural tissue changes that characterize cardiovascular damage in type 1 and type 2 diabetes (7).

### Demographic Factors

As for the general population, the absolute risk for ASCVD in diabetes increases with age in both type 1 and type 2 diabetes (8, 9) although the relative risk is highest in young adults and then falls with age. Women appear to lose their relative protection from CHD and stroke and have a greater relative risk compared to men but this falls as they age such that the prevalence of ASCVD becomes similar in elderly men and women with diabetes (10). Most minority groups have lower rates of ASCVD compared to Caucasians except for South Asians, a point that has been emphasized in a recent American College of Cardiology/American Heart Association Task Force on Clinical Practice Guidelines report (11), and socioeconomic status is associated with higher mortality in type 2 diabetes (12). While the basis for these differences in effects of demographic factors on ASCVD risk in diabetes is poorly understood, their clinical relevance is significant.

### Onset and Duration of Diabetes

ASCVD risk is related to duration of diabetes independent of aging (13) although it is confounded by age. Onset of diabetes is usually obvious in type 1 diabetes, particularly when this develops in children and adolescents in whom ASCVD is rare before age 30 years (14) but the onset of type 2 diabetes is more insidious and diabetes may be present for years before clinical diagnosis. Added to this the incidence of type 2 diabetes has been increasing in obese children and adolescents and it is likely that their ASCVD risk will be substantial in young and mid-adulthood (15) although definitive evidence is not yet available. Also uncertain is how much ASCVD risk is increased in newly diagnosed diabetes in the elderly. Underlying these considerations is our lack of understanding of the impact of the pre-clinical phase of diabetes on atherosclerosis. Depending upon how it is defined, up to 1 in 3 individuals have prediabetes and many of these individuals will develop type 2 diabetes (16). Hence the origin of accelerating atherogenesis likely begins early in the course of development of type 2 diabetes and there is evidence that people with prediabetes already have modestly increased ASCVD risk (17) thus offering an opportunity for intervention in this early, identifiable phase of type 2 diabetes.

### Risk Factors and Risk Enhancers

#### Major Risk Factors

The major ASCVD risk factors, hypercholesterolemia, cigarette smoking and hypertension are strongly related to development of ASCVD in diabetes as in the general population, although compared to non-diabetic subjects matched for these three risk factors, the incidence of CHD mortality remains 2-fold increased in diabetes, indicating the importance of other determinants of risk (18). Low density lipoprotein cholesterol (LDL-C) levels are similar in diabetes to those without diabetes, but the frequency of hypertension is ~2-fold increased in diabetes. Clinical management of these major risk factors together with treatment of hyperglycemia constitutes the basis for primary and secondary prevention of ASCVD in diabetes.

#### Hyperglycemia and Insulin Resistance

Much of the underlying substrate for ASCVD risk is likely to be related to hyperglycemia, insulin resistance and obesity and the accompanying pro-inflammatory and procoagulant states. The degree of hyperglycemia is related to CVD risk in populations without known diabetes (19) although the associations are attenuated after adjustment for other risk factors and this is true for obesity as well. More compelling in those with established diabetes is the evidence that improving hyperglycemia reduces ASCVD in clinical trials of anti-hyperglycemic agents as discussed below, although this is confounded by off-target effects of the antidiabetic medications. There is also evidence that insulin resistance is associated with ASCVD (20). However, this evidence is based mostly on epidemiologic assessments of insulin resistance which incorporate glucose values and are imperfect surrogate measures of insulin resistance—particularly in diabetes, and have generally not led to clinically useful risk assessment or intervention strategies with the possible exception of dyslipidemia.

#### Dyslipidemia—an Atherogenic Tetrad

Insulin resistance in type 2 diabetes is thought to be a key determinant of hypertriglyceridemia and reduced high density lipoprotein cholesterol (HDL-C), both common abnormalities in type 2 but not type 1 diabetes and they have been associated with ASCVD risk in type 2 diabetes (21). The HDL-C level is inversely and strongly related to ASCVD, and is included together with age, sex, total cholesterol, blood pressure (BP), and presence or absence of smoking in the risk factor algorithms used to quantify ASCVD risk in diabetes (22–24). Lack of success in clinical trials to raise HDL-C pharmacologically has led to the notion that the basis for the strong inverse association between HDL-C and ASCVD may be related to HDL dysfunctionality (25), which is not sufficiently captured by the HDL-C value in high risk states where HDL may be dysfunctional. In support of this concept, very high HDL-C was shown paradoxically to be a direct risk factor for ASCVD in type 1 diabetes (26) in whom HDL-C levels tend to be elevated (27). Triglyceride levels are a less powerful risk factor for ASCVD in diabetes, and triglyceride-lowering with pharmacologic agents has not been shown to be associated with a reduction of ASCVD events. More likely hypertriglyceridemia is a marker for other metabolic abnormalities such as dysfunctional HDL, atherogenic small dense LDL and remnant lipoprotein particles, making up an atherogenic tetrad (28, 29). Methods that efficiently quantify lipoprotein subfractions have demonstrated that selected subfractions are strongly correlated with insulin resistance and are currently in clinical use although it remains for them to be shown to be independent predictors of ASCVD in diabetes.

#### Risk Enhancers

The concept of risk enhancers was recently incorporated into risk assessment (24) to include factors that are not typically included in risk factor algorithms yet are sufficiently associated with ASCVD event rates to warrant consideration in risk assessment (Table 2). Of relevance to individuals with diabetes these include hypertriglyceridemia, elevated apolipoprotein B as a marker of increased numbers of atherogenic particles, and chronic kidney disease, which is common in diabetes due to development of diabetic nephropathy, manifesting as albuminuria or as reduced glomerular filtration rate. Subclinical tests of peripheral vascular and coronary artery disease such as the ankle brachial index obtained by Doppler ultrasound and the coronary calcium score measured by computerized tomography are strongly related to future occurrence of ASCVD in diabetes although their clinical utility is unclear. Lastly the presence of any form of diabetic microangiopathy whether it be retinopathy, peripheral neuropathy, or nephropathy are all associated with increased risk of ASCVD possibly because of common pathways for vascular damage (30–32) and should be considered in risk assessment.

**Table 2:** Risk enhancers for atherosclerotic cardiovascular disease (ASCVD) (14).

**Specific to Diabetes**	**General**
**RISK ENHANCERS**	
Long duration (≥10 years for type 2 diabetes mellitus or ≥20 years for type 1 diabetes mellitus	Family history of premature ASCVD
Albuminuria ≥30 mcg of albumin/mg creatinine	LDL-C levels >160 mg/dl
eGFR <60 mL/min/1.73 m^2^	Metabolic syndrome
Retinopathy	Chronic kidney disease
Neuropathy	History of preeclampsia or premature menopause in women
Ankle brachial index <0.9	Chronic inflammatory disorders
	High-risk ethnicity such as South Asian ancestry
	Triglyceride levels persistently >175 mg/dl
	If measured, elevations in apolipoprotein B (may be useful if hypertriglyceridemia >200 mg/dl persists
	High sensitivity C reactive protein >2 mg/L
	Lipoprotein(a) levels with elevations above 125 nmol/L (50 mg/dl) (especially useful in those with a family history of ASCVD)
	Reduced ankle brachial index

*eGFR, estimated glomerular filtration rate*.

## Recent Trends in the Prevalence of ASCVD in Diabetes

**Figure 1:**
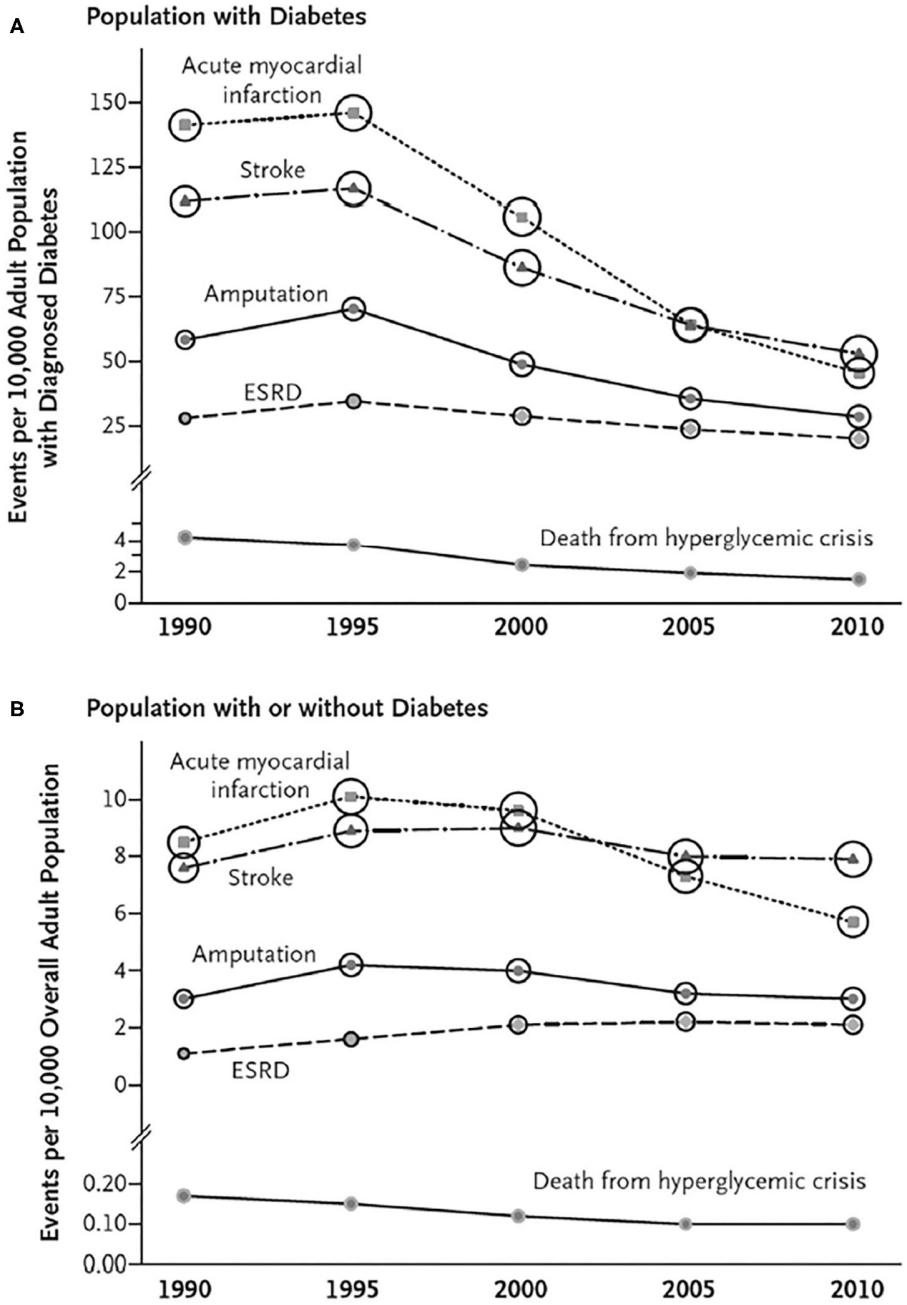
Trends in Age-Standardized Rates of Diabetes-Related Complications among U.S. Adults with and without Diagnosed Diabetes, 1990–2010. For rates of myocardial infarction, stroke, and leg amputation, numerators are from the National Hospital Discharge Survey; for rates of end-stage renal disease (ESRD), numerators are from the U.S. Renal Data System, and for rates of death from hyperglycemic crisis, numerators are from the National Vital Statistics System. Denominators are from the National Health Interview Survey **(A)** and the U.S. Census Bureau **(B)**. Circle size is proportional to the absolute number of cases (e.g., the number of cases of acute myocardial infarction ranges from 140,122 in 1990 to 135,743 in 2010, and the number of cases of death from hyperglycemic crisis ranges from 2890 in 1990 to 2361 in 2010). **(A)** Shows trends for persons with diabetes, and Panel B shows trends for persons with or without diabetes [From (33); with permission].

Data collected over the past three decades on the incidence of cardiovascular disease in diabetes indicates a significant decline in myocardial infarction (MI), stroke and leg amputation in the US and similar findings have been reported from other high income countries (Figure 1A) while this is not observed in the general population (Figure 1B) (34). Although this may have been influenced by earlier diagnosis of diabetes over time leading to an overall healthier population with diabetes, it is possible that improvements in management strategies that reduced ASCVD risk may have played a role. The fact that CVD event rates have been demonstrated to be strongly related to whether the LDL-C, BP and the glycosylated hemoglobin (HbA1c) level as a measure of glycemic control were at their respective targets supports this contention (35). In contrast to the fall in the incidence of occlusive atherosclerotic events, the incidence of HF with its attendant high morbidity and mortality has been increasing (36). Most cases are thought to be due to CHD, but in recent years it has become apparent that the combined effects of diabetes, obesity and aging cause cardiomyopathic changes leading to loss of left ventricular compliance and a form of HF (HF with preserved ejection fraction) that does not respond well to conventional therapies for HF with reduced ejection fraction that typically results from MI (37, 38).

These findings provide an incentive to initiate effective, evidence-based interventions in people with diabetes particularly in view of the fact that the mortality rate after a first event in those with diabetes is significantly increased compared to those without diabetes (39). The approaches to prevention of ASCVD in diabetes described below form the basis for similar recommendations from both United States and European society guidelines (22–24, 40).

## Approach to Prevention of ASCVD

### Weight Reduction

Despite the fact that intensive lifestyle modification achieving prolonged moderate weight loss and increased physical activity in type 2 diabetes has been shown in a controlled clinical trial to produce favorable changes in CVD risk factors, it did not lead to a reduction in major adverse cardiovascular events (MACE) over a 10 year period (41). Greater degrees of weight reduction are achieved by bariatric surgery and in a large controlled but non-randomized study in which the control group received standard diabetes and obesity management, the gastric bypass surgery group had fewer MI events but not stroke (42). In a more recent controlled randomized study, gastric bypass had significant and sizable benefit for HF and renal disease outcomes but not on MI or stroke suggesting that the benefits of weight reduction for diabetic complications are greatest for cardiac and renal dysfunction rather than for atherosclerotic events (43).

### Hyperglycemia

#### Sulfonylureas, Metformin, Insulin

The first definitive study to show that improving glycemic control in type 2 diabetes lowered the risk of complications, tested the effects of intensified treatment with the sulfonylurea insulin secretagogues as primary therapy to which insulin could be added, vs. standard care with diet and addition of sulfonylureas to treatment only to prevent severe hyperglycemia. While intensified treatment lowered the risk of microvascular complications over the 10 period, the effect on MI did not quite reach significance (44). Importantly though the effect of newly introduced metformin, which inhibits hepatic glucose overproduction through an effect on AMP kinase, did show a beneficial effect in a parallel smaller substudy, but not when combined with sulfonylurea drugs (45), raising questions about the use of sulfonylurea agents for prevention of ASCVD. However, long term follow-up of the original intensified treatment group during which the HbA1c levels in the intensive and standard groups were no longer different, found a significant reduction in MI events suggesting the existence of a legacy effect of improved glycemia that has been attributed to metabolic “memory” (46). Similar long-term follow-up findings were obtained after intensive insulinization in young adults with type 1 diabetes. Although there was no benefit on ASCVD during a 6.5 year period of intensive glycemic vs. standard control, after a further 12 years of follow-up when HbA1c values became similar in the two groups, total CVD events were reduced by 42% and MACE by 57% in the intensively treated group (47). These data in type 1 diabetes are the best evidence that improved glycemic control reduces ASCVD risk, because the two study groups received treatment with the same agent, namely insulin. There have been no equivalent studies with insulin only in type 2 diabetes.

#### Thiazolidinediones

The issue of possible deleterious off-target effects became a further concern after rosiglitazone, the first of the thiazolidinediones, which activate peroxisome proliferator activated ɤ-receptors (PPARɤ), was found to be associated with an increase in MI and CVD death (48). Subsequently pioglitazone, a thiazolidinedione with somewhat more favorable effects on CVD risk factors thought to be related to differences from rosiglitazone in binding to PPARɤ, was shown to have beneficial effects on MACE and especially stroke in type 2 diabetes (49, 50). However, both agents increase risk for HF, at least in part through water retention (51).

#### DPP4 Inhibitors, GLP-1 Agonists, and SGLT2 Inhibitors

More recently the newer antihyperglycemic agents namely the dipeptidyl peptidase 4 inhibitors (DPP-4i), the glucagon like peptide-1 agonists (GLP-1a) and the sodium/glucose transporter 2 inhibitors (SGLT2i) have all been tested in clinical trials for non-inferiority to standard treatments with pre-existing agents on ASCVD outcomes, as is now required for new antidiabetic agents by the US Food and Drug Association because of concern for deleterious off-target effects. Compared to therapy with older agents, DPP-4i had no effect on ASCVD events other than an increase in HF long-term attributable mainly to the SAVOR-TIMI trial with saxagliptin (52). However, clinical trials with GLP-1a have demonstrated that overall these agents modestly reduce MACE by 8% but not CVD death or HF (53). By contrast SGLT2i's reduced HF and CVD death by 24% in patients with pre-existing ASCVD, lowered recurrent ASCVD events by 14% events and decreased the worsening of renal disease by 26% (54). They are also fairly effective in lowering BP (55) which likely contributes to their beneficial effects. Furthermore, their benefit for cardiorenal outcomes especially HF resembles the findings noted after gastric bypass and points to possible common mechanisms that tie these two forms of therapy together in prevention of cardiorenal complications. Although there were small differences in HbA1c between the test and standard care groups in these studies, these were not found to account for the beneficial effects of the GLP-1a and SGLT2i and so these benefits are considered to be off-target protective cardiovascular effects though they are not well-understood (56).

#### Clinical Guidelines for ASCVD Reduction Through Glucose Lowering (Figure 2)

The studies with sulfonylurea, metformin, insulin, and the thiazolidinediones provide support for the recommendation that improvement of glycemic control has long term benefits on ASCVD risk in both type 1 and 2 diabetes although they did not point to a clear target for this treatment. In addition they raised questions about active treatment differences between sulfonylureas and metformin and with rosiglitazone that pointed to possible deleterious off-target effects on ASCVD. Subsequent large observational studies suggest that sulfonylurea agents are associated with a higher incidence of CVD and death than metformin (57) that has relevance given that these two drugs are still the most commonly used antidiabetic medications in type 2 diabetes in part because of their inexpensiveness. The possible deleterious effects of sulfonylurea agents may be due to their inhibition of pre-ischemic conditioning (58); for rosiglitazone the mechanism is unknown. More recently three large clinical trials using various combinations of available antidiabetic medications including insulin but with minimal use of GLP1a and SGLT2i compared intensive vs. standard glycemic treatment aimed at reaching HbA1c values below what has become the usually accepted HbA1c target of 7% as a measure of good glycemic control (59). They showed trends but no significant benefit for CHD events and in one of them there was actually an increase in mortality forcing the trial to be stopped. These trials also drew attention to the risks of hypoglycemia in sulfonylurea and insulin treated patients since its incidence was increased in these studies. Although it was unclear from these studies whether increased hypoglycemia contributed to the lack of benefit, prospective studies have demonstrated that severe hypoglycemia is accompanied by an increased risk of CHD (60). Overall, when these data were included in a meta-analysis with the earlier studies, an average reduction of HbA1c from 7.8–6.9% was associated with a significant reduction of 15% in CHD outcomes (61).

**Figure 2:**
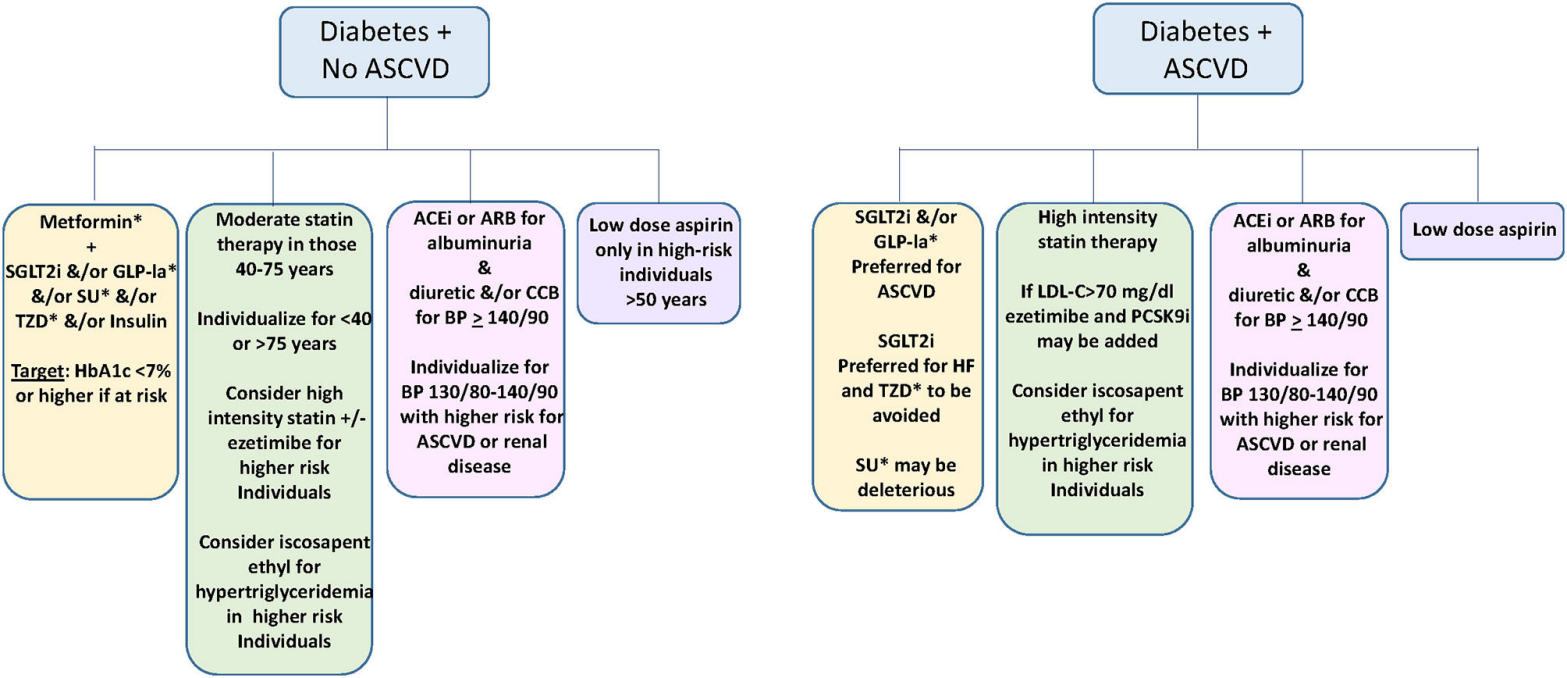
Suggested approach to medical prevention and amelioration of ASCVD in diabetes (22–24, 41). Yellow; Glycemic control. Green: Lipid management. Pink; Blood pressure management. Violet; Use of low dose aspirin. SGLT2i, sodium glucose transporter 2 inhibitor; GLP-1a, Glucagon like peptide 1 agonist; SU, sulfonylurea; TZD, thiazolidinedione; ACEi, Angiotensin converting enzyme inhibitor; ARB, angiotensin receptor blocker; CCB, calcium channel blocker; LDL-C, low density lipoprotein cholesterol. *not used in type 1 diabetes.

Based on the current evidence, GLP-1a and/or SGLT2i typically with metformin are favored for glycemic management for type 2 diabetes in those with established ASCVD and possibly in those with high risk for ASCVD, with the goal of achieving an HbA1c of <7% if this can be done safely (22, 23, 40).

### Dyslipidemia (Figure 2)

#### LDL-C

People with type 2 diabetes and a small number with type 1 diabetes in the 40–75 year age group were included in most of the placebo controlled trials with statins and benefitted in a similar manner to those without diabetes, although because of their higher ASCVD event rates, the absolute reduction in events was always greater in those with diabetes, both in primary and secondary prevention studies (62). There have been 3 primary prevention trials conducted in large cohorts with diabetes and average LDL-C levels, and another that recruited individuals with and without prior ASCVD (63). All used moderate intensity statin therapy which lowers LDL-C ~30% and overall they demonstrated that ASCVD relative risk was lowered 25% with no apparent difference in benefit between type 1 and 2 diabetes. This led to the recommendation that moderate intensity statin therapy is indicated for adults with diabetes aged 40–75 years. Assessment of ASCVD risk using quantitative risk assessment algorithms is not deemed necessary for this decision although these algorithms may be useful in refining risk assessment in individual patients. Furthermore, since the residual risk for ASCVD events remained in the intermediate risk range after moderate intensity statin-treated individuals, high intensity statin therapy which lowers LDL-C ~50% and which has been shown to lead to incremental benefit (62) is preferred for primary prevention in those with multiple risk factors as is recommended for patients with established ASCVD (22–24, 40).

Ezetemibe, an intestinal cholesterol absorption inhibitor, may be added to reach this goal if necessary in view of its incremental effectiveness when added to a statin (64). For secondary prevention in very high risk individuals into which category older patients with diabetes fall, an LDL-C target of <70 mg/dl has been proposed which may require the addition of inhibitors of propeptide convertase subtilisin/kexin 9 (PCSK9i). PCSK9i prevent the action of this protein to promote intracellular catabolism of the LDL receptor (22–24, 40) and like ezetimibe, PCSK9i have been shown to further reduce ASCVD events in high-risk statin treated individuals with diabetes in proportion to its additive LDL-C lowering (65, 66). Since there are very little or no data on the benefit of pharmacologic LDL-C lowering in people with diabetes below the age of 40 years or those older than 75 years, this decision is left to medical judgement based on perceived benefit vs. safety (24).

#### Triglyceride

Since hypertriglyceridemia and reduced HDL-C are common in type 2 diabetes despite dietary recommendations aimed at losing weight through reduction of refined carbohydrate and saturated fat, and likely contribute to ASCVD risk, triglyceride-lowering agents such as fibric acid derivatives and high dose omega 3 fatty acid preparations have been evaluated for their utility in preventing CVD in type 2 diabetes. These agents have been largely unsuccessful in demonstrating benefit for ASCVD in placebo-controlled clinical trials although they have generally not been specifically tested in hypertriglyceridemic subgroups with diabetes. Secondary analyses from the fibrate trials have suggested possible benefit for fibrate therapy in those with triglyceride values >200 mg/dl and HDL-C levels <35 mg/dl (67). In a recent clinical trial, icosapent ethyl (68), a synthetic derivative of the omega 3 fatty acid eicosapentaenoic acid was compared to placebo in a large statin-treated cohort either with CHD or type 2 diabetes without ASCVD and one risk factor, and with triglyceride levels >135 mg/dl. There was a 25% relative risk reduction in ASCVD events including on CVD death unrelated to the amount of triglyceride lowering achieved, suggesting the benefit was related to other effects of the specific omega 3 fatty acid used. This agent is now being recommended for high risk individuals with diabetes on statin treatment with residual hypertriglyceridemia (24). Trials with apo C-III, and angiopoioetin-like-3 antisense oligonucleotides are yielding promising results for treatment of hypertriglyceridemia that may yield benefit for ASCVD risk (69, 70). In addition studies with an antisense oligonucleotide nucleotide to lipoprotein (a) may become important in reducing risk related to this risk enhancer (71).

### Hypertension (Figure 2)

Controlled clinical trials have clearly demonstrated that lowering BP to <140/90 reduces the risk of both microvascular and ASCVD complications in cohorts with diabetes (23). Reducing weight and lowering sodium intake lowers blood pressure but is usually insufficient. Pharmacologic treatment should begin with any of either an angiotensin converting enzyme inhibitor (ACEi) or angiotensin receptor blocker (ARB), a calcium channel blocker, or a diuretic all of which individually have been shown to reduce ASCVD events in clinical trials mostly conducted in cohorts with large diabetes subgroups. For those with albuminuria or CKD, agents that reduce intraglomerular pressure such as an ACEi or ARB are favored because of their specific benefits for progression of renal disease (22–24) but many patients require multidrug therapy. Since the association between BP and ASCVD risk begins at values below 140/90, several clinical trials have tested more intensive treatment aimed at achieving lower BP targets. Overall there may be additional benefit for stroke and microvascular disease outcomes but not clearly for CHD events in those with diabetes, and there was an increased likelihood of drug side-effects, so one recommendation is to treat to a target of 140/90 with individualization for more intensive treatment to 130/80 in individuals with higher risk such as those with established ASCVD and renal disease (22, 23). Others have proposed more uniform treatment to a target of 130/80 in people with diabetes (40).

### Aspirin (Figure 2)

Low dose aspirin's antiplatelet effect has been shown to be effective in reducing MACE. The relative risk reduction is about 25%, and stronger for MI than ischemic stroke but the risk of serious hemorrhagic complications particularly in the elderly although small, is a significant safety concern especially in primary prevention where the absolute risk for ASCVD events is considerably less than in those with established CVD. Accordingly while low dose aspirin is recommended to prevent recurrent ASCVD in diabetes, use of aspirin in primary prevention is proposed for those with diabetes in the 50–70 year age range who have at least one additional risk factor for ASCVD including renal disease (23, 24, 40).

### Anti-inflammatory Agents

In a placebo-controlled clinical trial in a statin treated cohort with elevated high sensitivity C reactive protein level as a measure of subclinical inflammation with a past history of MI, canakinumab, a monoclonal antibody to interleukin 1Lβ, reduced MACE by 15% in the mid-range dose although there was a higher incidence of fatal infections (72). Forty percent of the cohort had diabetes and the relative risk reduction in this subgroup was 10% which did not reach significance. Although not ready for clinical use, this study provides clinical evidence for the concept that inhibiting a pathway of inflammation may reduce ASCVD events. A subsequent trial with methotrexate an immunosuppressant and inhibitor of IL-6 binding was not effective, while colchicine, a microtubule inhibitor with anti-inflammatory effects reduced total CVD events although this was only significant for coronary revascularization and stroke (73, 74).

## Conclusion

During the past 20 years significant advances have been made in understanding the relationship between clinically relevant risk factors in relation to age, sex and type and duration of diabetes and the augmentation of ASCVD in diabetes. These have led to the application of interventions targeting glycemic control, LDL-C lowering, BP lowering and the prothrombotic state that have demonstrated effectiveness in individual clinical trials to lower rates of ASCVD events. Incorporation of these findings into clinical guidelines has likely contributed to the fall in prevalence of MI, stroke and amputation in diabetes. Although there has been only one long-term controlled clinical trial evaluating the combined effects of multiple risk factor interventions on vascular complications in diabetes, it demonstrated a 53% reduction in CVD death and a 59% reduction in total CVD events over a 13.3 year follow-up (75). Widespread application of the guidelines combined with earlier diagnosis of diabetes together with newer developments of novel pharmacologic agents should strengthen and broaden efforts to improve quality of life and longevity in people with diabetes.

## Author Contributions

RG was solely responsible for preparing and writing the review.

## Conflict of Interest

The author declares that the research was conducted in the absence of any commercial or financial relationships that could be construed as a potential conflict of interest.
